# Supplementary material for: A neurophysiological basis for aperiodic EEG and the background spectral trend
Source: Nat Commun. 2024 Feb 19;15:1514. doi: 10.1038/s41467-024-45922-8 (PMC10876973; doi:10.1038/s41467-024-45922-8)
Supplement: Supplementary file 1 — Supplementary Information [file 41467_2024_45922_MOESM1_ESM.pdf]

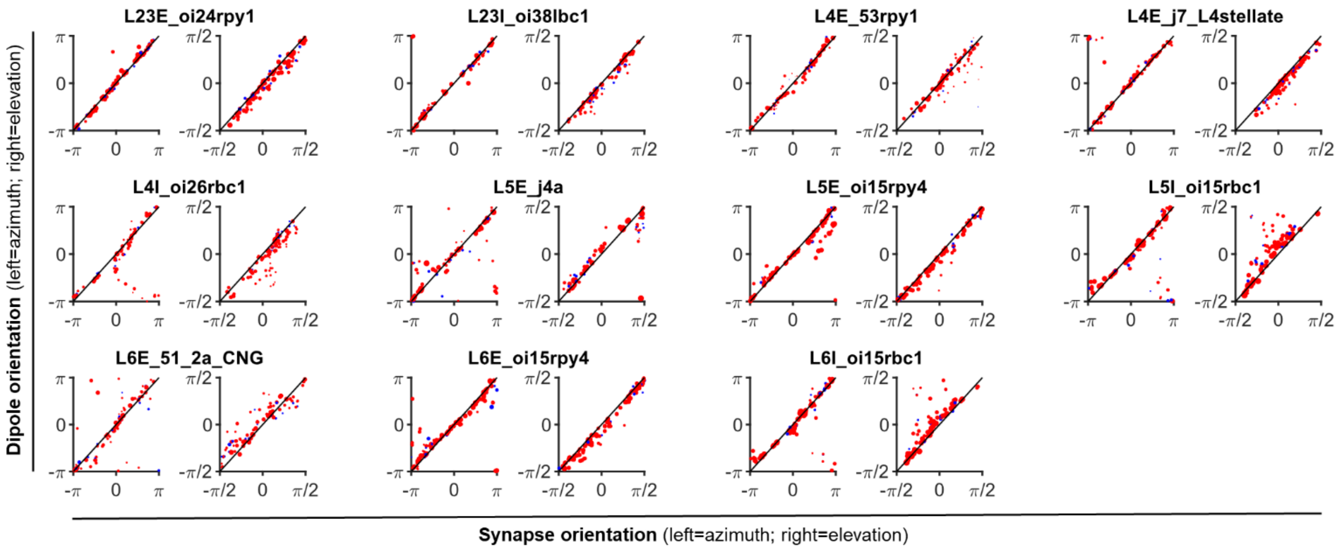

Supplementary Figure S1. Relationships between synapse orientation and dipole orientation.

Dipole moments were simulated following a single synapse activation at various locations across the 11 representative neuron morphologies (Table S1). For each morphology, two plots are shown: the left plots show the azimuth of the dipole moment plotted against that of the synapse that was activated; the right plots show the elevations. Red dots reflect excitatory synapses and blue dots reflect inhibitory synapses. Unity lines are drawn in black.

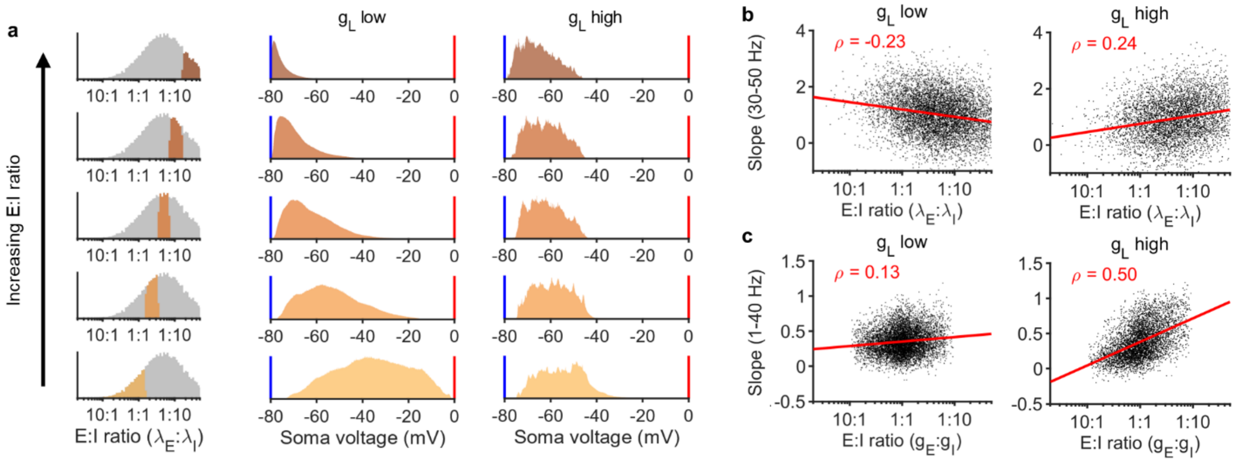

Supplementary Figure S2. Effects of excitatory-inhibitory ratio on membrane potential and spectral slope depend on leak conductance.

a Left: histogram of E:I ratios across 20,000 simulations with parameters sampled from the distributions in Fig. 6a. Simulations were binned into five categories, from low to high $\lambda_E : \lambda_I$ ratios. Middle: histogram of somatic membrane potential for simulations with a low leak conductance (g_L < 1 mS cm^{-2}), divided into the five E:I ratio categories. The average membrane potential is not largely affected by the E:I ratio in this high leak conductance condition. Right: histogram of somatic membrane potential for simulations with a high leak conductance (g_L > 1 mS cm^{-2}). A high E:I ratio significantly shifts the distribution of membrane potential to more hyperpolarized values. Blue and red vertical lines show the reversal potential of GABARs and AMARs, respectively.

b Same as Fig. 6e, but for the slope computed between 30-50 Hz for g_L high ($\rho = 0.24$; R^2=0.06; p<10^{-6}; n=5184 simulations) and g_L low ($\rho = -0.23$; R^2=0.05; p<10^{-6}; n=7366 simulations).

c Same as Fig. 6e, but with the E:I ratio defined as the ratio between g_E and g_I , for g_L high ($\rho = 0.50$; R^2=0.25; p<10^{-6}; n=5184 simulations) and g_L low ($\rho = 0.13$; R^2=0.02; p<10^{-6}; n=7366 simulations)

.

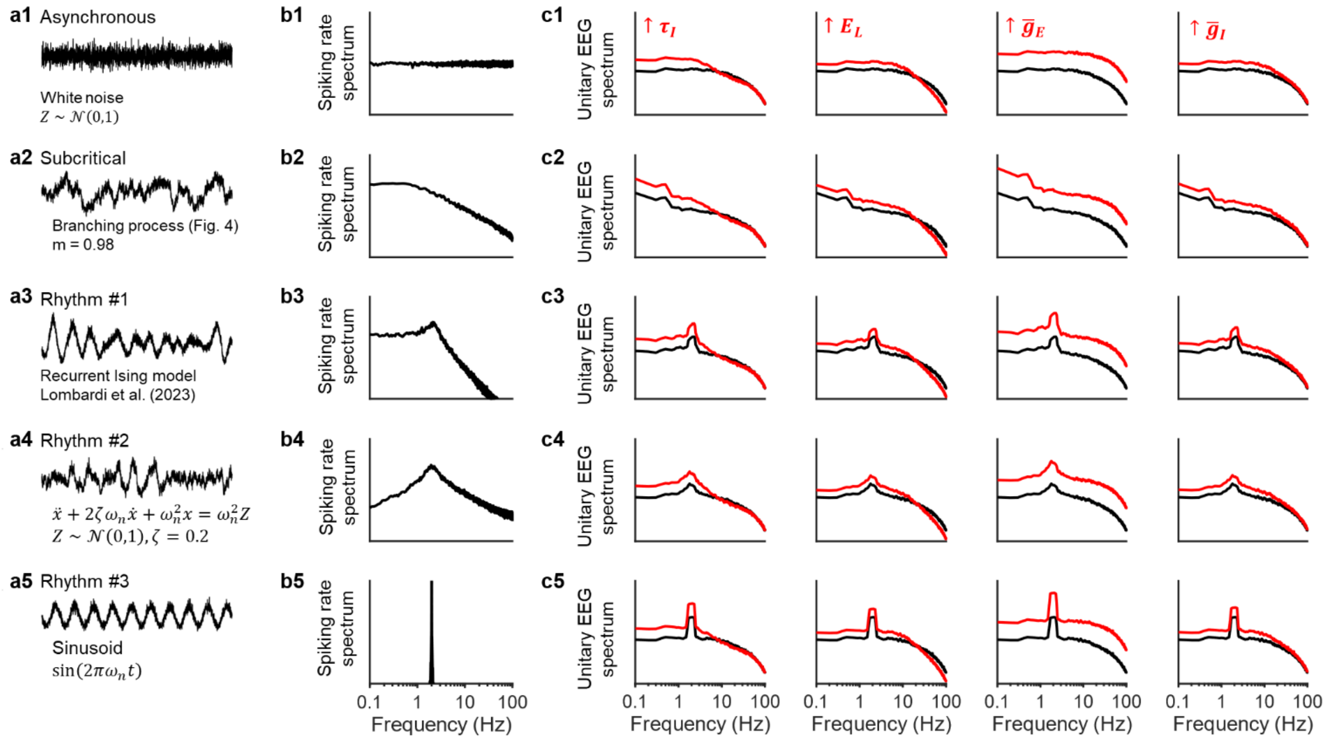

Supplementary Figure S3. Biophysical parameters alter broadband EEG properties across many types of dynamics.

a1-5 Five examples of synaptic input dynamics, generated by asynchronous white noise (**1**), a subcritical branching process (**2**), a recurrent Ising model[1] (**3**), an underdamped second-order linear system (**4**), and a sine wave (**5**). $\alpha_x = 0.1$ for all simulations in this figure.

b1-5 Power spectra of the rate functions for each type of synaptic input depicted in **a1-5**, respectively.

c1-5 Unitary spectra associated with input depicted in **a1-5**, before and after changes to a biophysical parameter, including τ_I that was increased from 10 ms (black) to 30 ms (red) in the first column, E_L that was increased from -60 mV (black) to -45 mV (red) in the second column, and g_E and g_I that were increased from 0.7 nS (black) to 1.4 nS (red) in the third and fourth columns, respectively.

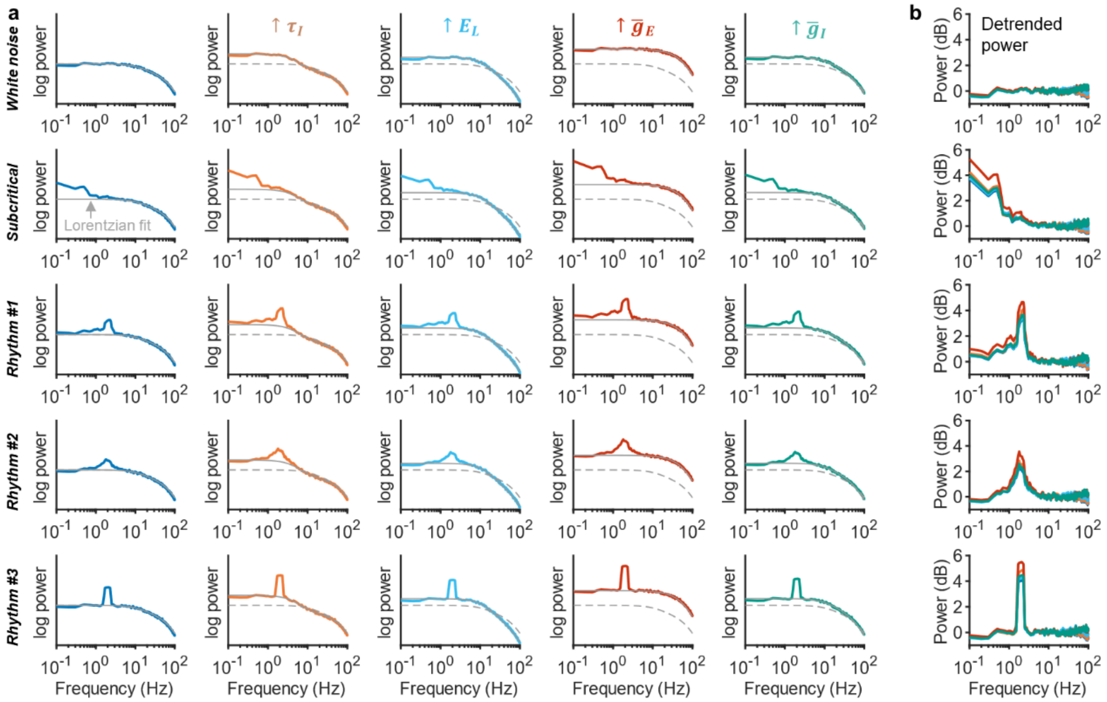

Supplementary Figure S4. Detrending with Lorentzian function corrects for changes in biophysical parameters.

a Unitary spectra from Fig. S3, fit with the sum of two Lorentzian functions (Eq. 1; solid gray lines). The leftmost column shows the unitary spectrum with default parameters. The Lorentzian fit for the default parameters are also displayed in the plots in the other columns as a dashed grey line.

b Unitary spectra from **a**, detrended by dividing by the fitted Lorentzian function (solid gray lines). Colours correspond to the various parameter changes from **a**. Note that, for each type of input dynamics, the detrended spectra have similar profiles, i.e., the effects of the parameter changes have been corrected.

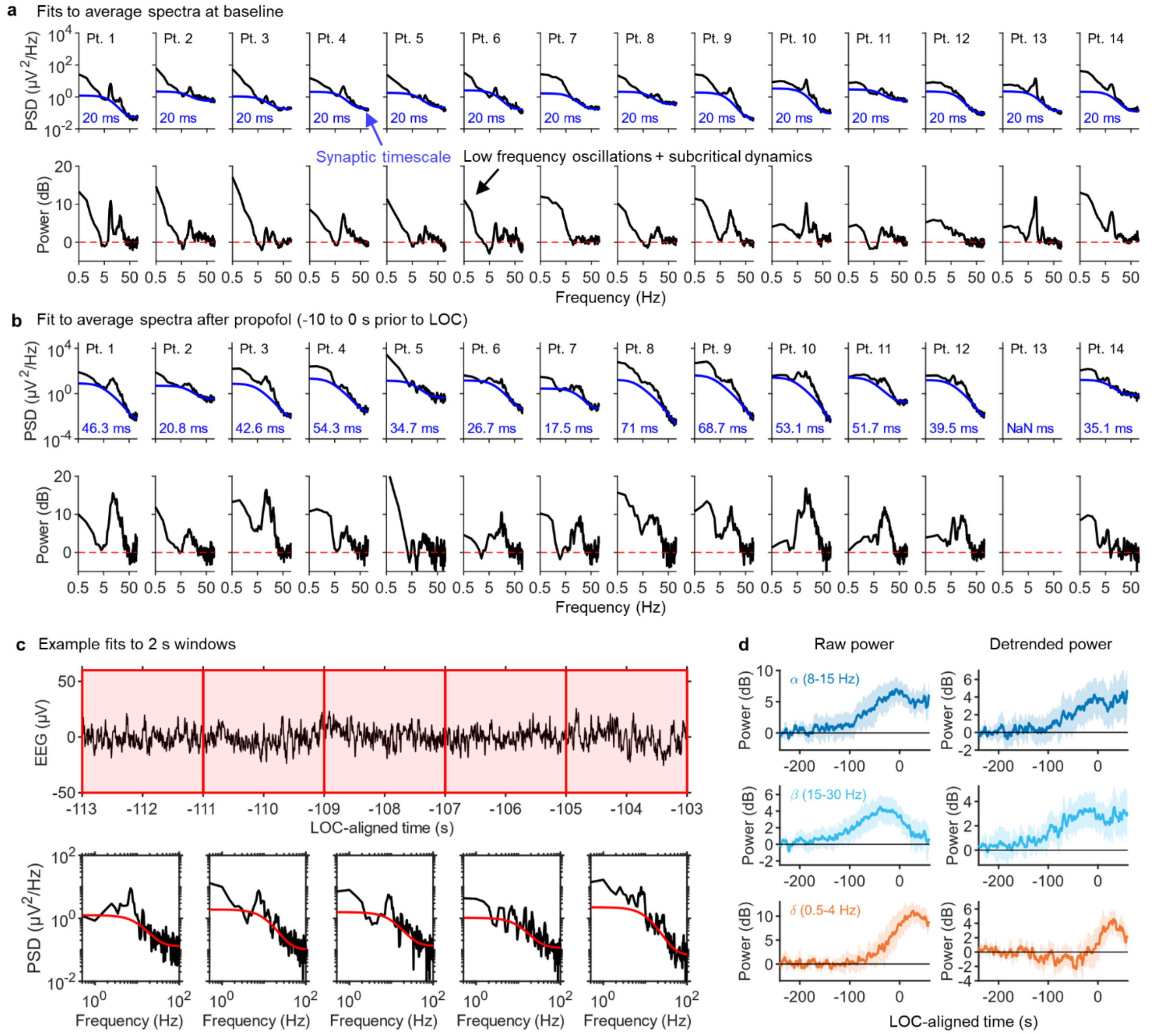

Supplementary Figure S5. Spectral changes with respect to LOC-aligned time.

a Top: example fits to the average EEG spectrum of each patient at baseline using Eq. 6, while fixing the parameters $\tau_r = 4$ ms and $\tau_1 = 20$ ms. Note the difference between the fitted Eq. 6 and the low frequency power (< 3 Hz), which our model predicts is caused by neural dynamics and not synaptic timescales. This low frequency power was fit here with a Gaussian peak function as per the FOOOF methods[2] (Gaussian fits not shown). Bottom: detrended power in decibels.

b Same as **a**, but for fits to spectra -10 to 0 s prior to LOC. Here, τ_1 was not fixed and its estimated value for each patient is printed in blue.

c Example EEG from patient 13, split into five, 2 s windows (top). The power spectrum of each window is shown below, along with the fitted synaptic timescales (Eq. 6) in red.

d Same as Fig. 9d & h, but band power is plotted here against time relative to LOC. Data plotted as mean and shading represents 95% confidence interval of mean.

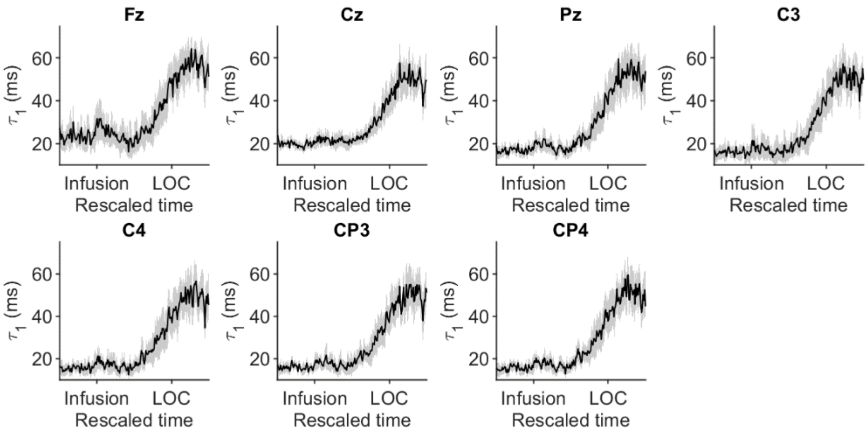

Supplementary Figure S6. Changes in estimated τ_1 exhibit similar dynamics across recording locations. The plot labelled Cz is identical to Fig. 8e. The other plots show the dynamics of τ_1 for the other EEG channels. For each plot, the corresponding recording site is printed above.

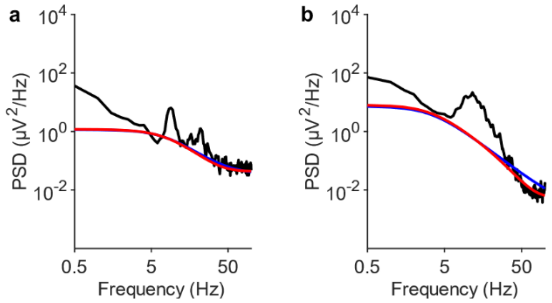

Supplementary Figure S7. Simple exponential decaying synaptic response does not capture spectral trend following propofol infusion.

a Example spectrum of subject 1 at baseline, same as in Fig. 8d. Blue line: data fitted with Eq. 5; $\tau_1 = 20$ ms, $A_1 = 56$, and $\lambda = 0.034$. Red line: data fitted with Eq. 6; $\tau_1 = 20$ ms, $\tau_2 = 4$ ms, $A_1 = 4470$, and $\lambda = 0.043$.

b Example spectrum of subject 1 following propofol infusion, 0 to 10 s prior to LOC, same as in Fig. 8d. Blue line: data fitted with Eq. 5; $\tau_1 = 46$ ms, $A_1 = 158$, and $\lambda = 0.0026$. Red line: data fitted with Eq. 6; $\tau_1 = 46$ ms, $\tau_2 = 4$ ms, $A_1 = 4570$, and $\lambda = 0.0051$. Note that the blue line is not steep enough to capture the drop-off in power around 30 Hz.

Supplementary Table S1. Representative neuron morphologies used in the model[a].

Cell type	Abundance (%)	Internal ID	External IDs[b]	Reference
Layer 2/3 pyramidal cell	26.8	L23E_oi24py1	NMO_10045	Ref. 3
Layer 2/3 interneuron	7.5	L23I_oi38lbc1	-	Ref. 4
Layer 4 excitatory stellate cell	19.0	L4E_j7_L4stellate	NMO_00905	Ref. 5
Layer 4 pyramidal cell	9.5	L4E_53rpy1	NMO_10040	Ref. 3
Layer 4 interneuron	7.1	L4I_oi26rbc1	-	Ref. 4
Layer 5 pyramidal cell	4.9	L5E_oi15rpy4	NMO_10046	Ref. 3
Layer 5 interneuron	1.4	L5I_oi15rbc1	-	Ref. 4
Layer 5 tufted pyramidal cell	1.3	L5E_j4a	MDB_2488	Ref. 5
Layer 6 excitatory cell	14.0	L6E_51_2a_CNG	NMO_00879	Ref. 6
Layer 6 pyramidal cell	4.6	L6E_oi15rpy4	-	Ref. 3
Layer 6 interneuron	3.8	L6I_oi15rbc1	-	Ref. 4

[a]Morphologies and relative abundances were identical to Hagen et al.[7].

[b]Morphologies without NeuorMoprho (NMO) or ModelDB (MDB) IDs were accessed from the code repository associated with Hagen et al.[7]. All morphology files are also supplied in the code repository associated with this paper[8].

Supplementary Table S2. In vitro data on inhibitory synapse kinetics in the presence of propofol.

	Study #1[a]			Study #2[b]					Study #3[c]						Studay #4[d]					
Propofol (μM)	0	0.5	2	0.1	0.3	1	3	10	0	0.5	1	2	5	10	0	0.5	1	2	5	10
τ (ms)	19.4	29.6	44.5	N/A	N/A	N/A	N/A	N/A	17.5	17.5	19	48	58	75	17.5	17.5	19	22.5	50	75
τ (fold change)	1	1.5	2.3	1	1.2	1.4	1.5	2	1	1	1.1	2.7	3.3	4.3	1	1	1.1	1.3	2.9	4.3

[a] Inhibitory post-synaptic current (IPSC) decay time constant taken from Fig. 8 of Orser et al.[9].
[b] Fold change in spontaneous IPSCs estimated from Fig. 7C of Kitamura et al.[10].
[c] Decay time of IPSCs during slow parts of evoked trains, taken from Fig. 5 of Whittington et al.[11].
[d] Decay time of IPSCs during fast parts of evoked trains, taken from Fig. 5 of Whittington et al.[11].

Supplementary References

1. Lombardi, F., Pepić, S., Shriki, O., Tkačik, G. & De Martino, D. Statistical modeling of adaptive neural networks explains co-existence of avalanches and oscillations in resting human brain. *Nat. Comput. Sci.* **3**, 254–263 (2023).
2. Donoghue, T. *et al.* Parameterizing neural power spectra into periodic and aperiodic components. *Nat. Neurosci.* **23**, 1655–1665 (2020).
3. Budd, J. M. L. *et al.* Neocortical Axon Arbors Trade-off Material and Conduction Delay Conservation. *PLoS Comput. Biol.* **6**, e1000711 (2010).
4. Stepanyants, A. *et al.* Local potential connectivity in cat primary visual cortex. *Cereb. Cortex* **18**, 13–28 (2008).
5. Mainen, Z. F. & Sejnowski, T. J. Influence of dendritic structure on firing pattern in model neocortical neurons. *Nature* **382**, 363–366 (1996).
6. Contreras, D., Destexhe, A. & Steriade, M. Intracellular and computational characterization of the intracortical inhibitory control of synchronized thalamic inputs in vivo. *J. Neurophysiol.* **78**, 335–350 (1997).
7. Hagen, E. *et al.* Hybrid scheme for modeling local field potentials from point-neuron networks. *Cereb. Cortex* **26**, 4461–4496 (2016).
8. Brake, N. niklasbrake/EEG_modelling: Editorial revisions (v1.1.0). Zenodo. https://doi.org/10.5281/zenodo.10607600 (2024).
9. Orser, B. A., Wang, L. Y., Pennefather, P. S. & MacDonald, J. F. Propofol modulates activation and desensitization of GABA(A) receptors in cultured murine hippocampal neurons. *J. Neurosci.* **14**, 7747–7760 (1994).
10. Kitamura, A., Marszalec, W., Yeh, J. Z. & Narahashi, T. Effects of halothane and propofol on excitatory and inhibitory synaptic transmission in rat cortical neurons. *J. Pharmacol. Exp. Ther.* **304**, 162–171 (2003).
11. Whittington, M. A., Jefferys, J. G. R. & Traub, R. D. Effects of intravenous anaesthetic agents on fast inhibitory oscillations in the rat hippocampus in vitro. *Br. J. Pharmacol.* **118**, 1977–1986 (1996).
