# Supplementary material for: A neurophysiological basis for aperiodic EEG and the background spectral trend
Source: Nat Commun. 2024 Feb 19;15:1514. doi: 10.1038/s41467-024-45922-8 (PMC10876973; doi:10.1038/s41467-024-45922-8)
Supplement: Supplementary file 3 — Reporting Summary [file 41467_2024_45922_MOESM3_ESM.pdf]

nature portfolio

Corresponding author(s): Anmar Khadra

Last updated by author(s): Dec 11, 2023

Reporting Summary

Nature Portfolio wishes to improve the reproducibility of the work that we publish. This form provides structure for consistency and transparency in reporting. For further information on Nature Portfolio policies, see our Editorial Policies and the Editorial Policy Checklist.

Statistics

For all statistical analyses, confirm that the following items are present in the figure legend, table legend, main text, or Methods section.

n/a	Confirmed	
☐	☒	The exact sample size (*n*) for each experimental group/condition, given as a discrete number and unit of measurement
☐	☒	A statement on whether measurements were taken from distinct samples or whether the same sample was measured repeatedly
☐	☒	The statistical test(s) used AND whether they are one- or two-sided<br>*Only common tests should be described solely by name; describe more complex techniques in the Methods section.*
☒	☐	A description of all covariates tested
☒	☐	A description of any assumptions or corrections, such as tests of normality and adjustment for multiple comparisons
☐	☒	A full description of the statistical parameters including central tendency (e.g. means) or other basic estimates (e.g. regression coefficient) AND variation (e.g. standard deviation) or associated estimates of uncertainty (e.g. confidence intervals)
☐	☒	For null hypothesis testing, the test statistic (e.g. *F*, *t*, *r*) with confidence intervals, effect sizes, degrees of freedom and *P* value noted<br>*Give P values as exact values whenever suitable.*
☒	☐	For Bayesian analysis, information on the choice of priors and Markov chain Monte Carlo settings
☒	☐	For hierarchical and complex designs, identification of the appropriate level for tests and full reporting of outcomes
☒	☐	Estimates of effect sizes (e.g. Cohen's *d*, Pearson's *r*), indicating how they were calculated

Our web collection on statistics for biologists contains articles on many of the points above.

Software and code

Policy information about availability of computer code

Data collection	Harmony ver.5.1 (Stellate Systems, Montreal) running on Windows XP.
Data analysis	All code used to analyze data is publicly available at github.com/niklasbrake/EEG_modelling (https://doi.org/10.5281/zenodo.10359818). The following Python packages were used to simulate our model:<br>LFPy==2.3<br>Cython==0.29.25<br>h5py==3.6.0<br>LFPykit==0.5.1<br>neuron==8.0<br>numpy==1.23.4<br>scipy==1.9.3<br>umap-learn==0.5.3<br>numba==0.56.3<br>pynndescent==0.5.8<br>scikit-learn==1.1.3<br>tqdm==4.64.1

For manuscripts utilizing custom algorithms or software that are central to the research but not yet described in published literature, software must be made available to editors and reviewers. We strongly encourage code deposition in a community repository (e.g. GitHub). See the Nature Portfolio guidelines for submitting code & software for further information.

Data

Policy information about availability of data

All manuscripts must include a data availability statement. This statement should provide the following information, where applicable:

- Accession codes, unique identifiers, or web links for publicly available datasets
- A description of any restrictions on data availability
- For clinical datasets or third party data, please ensure that the statement adheres to our policy

Computed EEG spectra have been deposited to figshare (https://doi.org/10.6084/m9.figshare.24777990), as well as all other files required to reproduce our manuscript figures.

Research involving human participants, their data, or biological material

Policy information about studies with human participants or human data. See also policy information about sex, gender (identity/presentation), and sexual orientation and race, ethnicity and racism.

Reporting on sex and gender	Sex and gender were not considered in study design. Gender information was not collected. Sex of the study subjects was recorded (10 males and 4 females); however, due to the low sample size of females, no post-hoc sex-based analysis was conducted. Individual-level data is provided in the Source Data file.
Reporting on race, ethnicity, or other socially relevant groupings	No socially relevant groupings were considered in our study and no such variables are reported in our manuscript.
Population characteristics	The population consisted of American Society of Anesthesiologists (ASA) class I or II patients presenting for lumbar disk surgery. 10 males and 4 females. 12 right-handed. The age and BMI of participants were 47.0 ± 11.1 years and 26.5 ± 3.2 kg per m^2.
Recruitment	We recruited subjects for the study from patients presenting for lumbar disk surgery; we do not expect EEG generation to be affected by the subject's lumbar afflictions or decision to participate in the study.<br><br>All patients (18-65 years old) scheduled for lumbar disk surgery were screened for eligibility by reviewing their pre-operative medical assessment. Exclusion criteria were: 1- any form of inability to consent, including mental illness, language problems, etc.; 2- any condition that would impair ability to perform handgrip; 3-important cardiovascular or pulmonary disease; 4- anticipated difficult tracheal intubation,5- any medical condition that could render a moderately slow induction of anesthesia inappropriate including gastro-esophageal reflux, anticipated difficult mask ventilation, obesity (BMI=>30) and obstructive sleep apnea. About a third of the patients were ineligible and the most common causes were obesity and obstructive sleep apnea, conditions often present together.<br><br>Patients deemed eligible were contacted by phone a few days before the scheduled surgery to validate the information obtained from the pre-operative assessment and to solicit their participation to the study. The vast majority of patients (90%) were interested in participating. The consent form was then by sent to them by email to give them ample time to review the proposed study and to decide whether to participate or not. All patients except one agreed to participate.<br><br>The two factors that could have had an impact on the results are obesity and obstructive sleep apnea. Drug kinetics are influenced by obesity. Obstructive sleep apnea is associated with day-time somnolence, which could have confounded the results. Since patients with either of these conditions were excluded, we do not see any self-selection or other biases that could have influenced the results.
Ethics oversight	Montreal Neurological Hospital Ethic Board

Note that full information on the approval of the study protocol must also be provided in the manuscript.

Field-specific reporting

Please select the one below that is the best fit for your research. If you are not sure, read the appropriate sections before making your selection.

☒ Life sciences ☐ Behavioural & social sciences ☐ Ecological, evolutionary & environmental sciences

For a reference copy of the document with all sections, see nature.com/documents/nr-reporting-summary-flat.pdf

Life sciences study design

All studies must disclose on these points even when the disclosure is negative.

Sample size	For studies examining the effects of anesthetics on the EEG, we followed the empirical rule that a sample size between 10-15 is sufficient to identify relevant findings i.e. of sufficient magnitude and constancy for eventual clinical applications. Below is a list of articles about mechanisms of anesthesia in human subjects and published in the two most prominent anesthesiology journals. The sample size ranges from

8 to 16.

Plourde, G et al. Attenuation of the 40-Hertz Auditory Steady State Response by Propofol Involves the Cortical and Subcortical Generators, Anesthesiology 2008; 108:233–42 (N=11)

Lee, H et al. Reconfiguration of Network Hub Structure after Propofol-induced Unconsciousness Anesthesiology 2013; 119:1347-59 (N=10)

Malekmohammadi, M et al. Propofol-induced Changes in α-β Sensorimotor Cortical Connectivity ,Anesthesiology 2018; 128:305-16) (N=12)

Sleigh, J et al. Electroencephalographic slow wave dynamics and loss of behavioural responsiveness induced by ketamine in human volunteers Br J Anaesth 123 (5): 592-600 (2019) (n=15)

Liang, Z et al. Information Integration and Mesoscopic Cortical Connectivity during Propofol Anesthesia, Anesthesiology 2020; 132:504–24 (N=9)

Pullon, R et al. Granger Causality of the Electroencephalogram Reveals Abrupt Global Loss of Cortical Information Flow during Propofol-induced Loss of Responsiveness, Anesthesiology, 2020; 133:774–86 (N=16)

Huang, Y et al.Dynamic changes in rhythmic and arrhythmic neural signatures in the subthalamic nucleus induced by anaesthesia and tracheal intubation Br J Anaesth 125 (1): 67-76 (2020) (N=12)

Data exclusions

Two participants were excluded because of failure to comply with the instructions during induction of anesthesia. One kept talking, the other kept moving their dominant arm.

Replication

No replication was done. We verified the validity of the data for each subject by:
1- confirming that the baseline EEG had normal characteristics by visual inspection of the time-domain traces and of the spectra;
2- confirming that the typical changes that are consistently seen after the administration of general anesthesia did indeed occur, based on visual inspection of the time-domain traces and of the spectra.

We initiated a related study using finger tapping instead of handgrip, using same anesthetic protocol. This could have provided some form of replication. But patient recruitment became more difficult because higher priority was progressively given to complex cases (fusion with instrumentation) by the spine surgery service. Patients requiring complex spine surgery are not appropriate for the study. They are in general older and have significant associated medical conditions. The number of potential candidates for the study decreased markedly. We are looking for other sites for recruitment.

Randomization

Randomization was not relevant to our study, because there was only a single experimental group. The variable of interested (EEG) was measured continuously before, during, and after the administration of propofol to a single group of participants.

Blinding

Investigator blinding was not relevant to our study, because all participants received the same treatment.

Reporting for specific materials, systems and methods

We require information from authors about some types of materials, experimental systems and methods used in many studies. Here, indicate whether each material, system or method listed is relevant to your study. If you are not sure if a list item applies to your research, read the appropriate section before selecting a response.

Materials & experimental systems

n/a	Involved in the study
☒	☐ Antibodies
☒	☐ Eukaryotic cell lines
☒	☐ Palaeontology and archaeology
☒	☐ Animals and other organisms
☒	☐ Clinical data
☒	☐ Dual use research of concern
☒	☐ Plants

Methods

n/a	Involved in the study
☒	☐ ChIP-seq
☒	☐ Flow cytometry
☒	☐ MRI-based neuroimaging

Plants

Seed stocks	N/A
Novel plant genotypes	N/A
Authentication	N/A
